# Down-regulation of miR-30b-5p protects cardiomyocytes against hypoxia-induced injury by targeting Aven

**DOI:** 10.1186/s11658-019-0187-4

**Published:** 2019-11-21

**Authors:** Lanfang Zhang, Xinwei Jia

**Affiliations:** Department of Cardiology, Affiliated Hospital of Hebei University, No. 212 Yuhua East Road, Baoding, 071000 Hebei People’s Republic of China

**Keywords:** Myocardial infarction, Hypoxia-induced injury, miR-30b-5p, Aven

## Abstract

**Background:**

Ischemia/hypoxia-induced cardiomyocyte apoptosis has been considered as a main cause of myocardial infarction. Here, we aimed to investigate the functional role of miR-30b-5p in hypoxic cardiomyocytes.

**Methods:**

AC16 human cardiomyocytes were cultured under hypoxia to simulate myocardial infarction. A qRT-PCR assay was performed to determine miR-30b-5p expression in hypoxic cardiomyocytes. Cell survival, injury and apoptosis were assessed by MTT, lactate dehydrogenase (LDH) release, and flow cytometry assays, respectively. The target gene of miR-30b-5p in hypoxic cardiomyocytes was validated by luciferase reporter assay and Western blotting.

**Results:**

MiR-30b-5p expression was found to be significantly upregulated in hypoxic AC16 cells. The in vitro experiments showed that downregulation of miR-30b-5p effectively alleviated hypoxia-induced cardiomyocyte injury. Furthermore, Aven is a potential target gene of miR-30b-5p and its downregulation could partially reverse the influence of miR-30b-5p knockdown on AC16 cells under hypoxia.

**Conclusions:**

Inhibition of miR-30b-5p could protect cardiomyocytes against hypoxia-induced injury by targeting Aven.

## Background

Myocardial infarction is one of the leading causes of morbidity and mortality worldwide and has become a health issue [1]. It is estimated that at least 1 million cases of myocardial infarction each year are diagnosed in the United States [2]. The criteria used to diagnose myocardial infarction are a serious of clinical features, including electrocardiographic interpretation, cardiac biomarkers of necrosis, pathology, and imaging [3]. It is known that cardiomyocytes can produce ATP to sustain proper contractile work and ionic homeostasis through oxidative phosphorylation [4]. An attenuated or reduced supply of oxygen is considered to be a potential causative factor for myocardial ischemia, leading to a series of pathological changes [4]. In particular, programmed cardiomyocyte death, as the major form of myocardial damage, can exacerbate and accelerate injury in myocardial infarction due to the occluded epicardial coronary artery undergoing hypoxia [5]. However, the molecular mechanisms underlying hypoxia-induced cardiomyocyte damage remain largely unclear.

As a class of small non-coding RNAs, microRNAs (miRNAs) could negatively regulate their target gene expression through binding their 3′-untranslated region (3′-UTR) participating in multiple biological events, including proliferation, differentiation, development, and cell apoptosis [6, 7]. Recent studies have indicated that aberrant expression of miRNAs is involved in human cardio-cerebrovascular disease [8], including myocardial infarction [9]. For instance, miR-24 is a key regulator in vascularity and cardiac fibrosis in myocardial infarction [10, 11]. In post-infarction, miR-99a plays an important role in cardioprotection via retarding heart remodeling, as well as improving cardiac function and cell survival through regulating cell apoptosis and autophagy [12]. As a member of miRNAs, miR-30b-5p was first identified as a tumor suppressor in gastric cancer [13] and colorectal cancer [14]. Afterwards, He et al. [15] found that miR-30b-5p plays an important role in cardiac hypertrophy by targeting CaMKIIδ. Emerging evidence further indicates that miR-30b was implicated in hypoxia/reoxygenation and homocysteine-induced apoptosis in H9C2 and coronary artery endothelial cells, respectively [16, 17]. Moreover, miR-30b negatively regulates autophagy through inhibiting Atg12-Atg5 conjugate in hepatic ischemia-reperfusion [18]. These facts might suggest a crucial role of miR-30b-5p in hypoxia-induced cardiomyocyte injury.

Aven is an anti-apoptotic protein that controls apoptosis partially by abrogating caspase activation through binding to Bcl-xL and Apaf-1 [19]. The expression profile of Aven has been found to be associated with exogenous erythropoietin and methylprednisolone in heart tissue after traumatic brain injury [20]. Based on this evidence, this study was designed to investigate the possible role of miR-30b-5p in regulating hypoxia-induced cardiomyocyte injury. We further evaluated whether Aven was a functional regulator involved in miR-30b-5p regulating hypoxia-induced cardiomyocyte injury.

## Materials and methods

### Cell culture and treatment

The human cardiomyocyte line AC16 was purchased from American Type Culture Collection (Manassas, VA, USA). AC16 cells were cultured in Dulbecco’s Modified Eagle’s Medium (DMEM, Gibco, NY, USA) with 10% fetal bovine serum (FBS), 1% penicillin and 100 μg/mL streptomycin (all from Invitrogen, CA, USA) and maintained in a humidified incubator containing 5% CO_2_ and 95% air at 37 °C as the normoxic condition. To imitate the myocardial ischemia, cells were incubated in a hypoxic condition with 94% N_2_, 5% CO_2_, and 1% O_2_ for 6, 12 and 24 h, respectively to stimulate different degrees of hypoxia.

### Cell transfection

The miR-30b-5p inhibitor (AGAACAGUGAAAUUUCCAGUCC) and the corresponding scrambled negative controls (NC) were provided by GenePharma (Shanghai, China). Small interfering RNA targeting Aven (siAven) and its corresponding siNC were designed and synthesized by Invitrogen. After conventional culture for 24 h in six-well plates, the above oligonucleotides were transfected into AC16 cells with the transfection reagent Lipofectamine 2000, followed by hypoxia treatment for 12 h.

### Quantitative real-time PCR (qRT-PCR)

For analyzing miR-30b-5p expression, miRNAs were isolated the cultured AC16 cells using a miRNeasy Mini kit. MiR-30b-5p expression was determined on a 7500 Fast Real-Time PCR system (Applied Biosystems, Carlsbad, CA, USA) using the TaqMan MicroRNA Assays kit. For the Aven expression assay, total RNA was extracted using Trizol (Invitrogen) and the SYBR Green PCR Kit (Invitrogen) was used to perform qRT-PCR assay with the following primer sequences: miR-30b-5p forward: 5′-ACGGGCAAAAATACTCCAGCTCTCAAT-3′, miR-30b-5p reverse: 5′-CTCTGGAAAACTGGTGTCGACTGGTGTC-3′; U6 forward: 5′-ATTGGAACGATACAGAGAAGATT-3′, U6 reverse: 5′-GGAACGCTTCACGAATTTG-3′; Aven forward: 5′-TTATGGTGGGCAGGTTGT-3′, Aven reverse: 5′- GCTGGATTGGCATTTGAA-3′; β-actin forward: 5′-GAACCCTAAGGCCAAC-3′, β-actin reverse: 5′-TGTCACGCACGATTTCC-3′. The relative gene expression levels were analyzed with the 2^-ΔΔCt^ method with U6 and β-actin as the internal controls for miR-30b-5p and Aven, respectively. Each sample was independently analyzed three times.

### MTT assay

Cell proliferation was estimated by MTT (Sigma-Aldrich) according to the manufacturer’s protocols. In brief, AC16 cells were seeded in a 96-well plate at a density of 5 × 10^3^ cells per well for 1, 2, 3, 4 and 5 days, respectively. Then, cells in each were incubated at 37 °C with 50 μl of MTT (5 mg/ml) in phosphate buffered saline (PBS) for 4 h. After removing cell supernatant, the reaction was terminated by addition of 200 μl of DMSO. Finally, a microplate reader (Bio-Rad, Hercules, CA, USA) was used to read the optical density (OD) value at 595 nm.

### Lactate dehydrogenase (LDH) assay

Cellular injury was monitored with a permeability assay based on the amount of lactate dehydrogenase (LDH) released from cell lysis in the supernatant using the LDH-Cytotoxicity Assay Kit (BioVision, Milpitas, CA). Briefly, 0.2% Triton X-100 (Sigma-Aldrich) was used to lyse cells. After centrifugation, we harvested supernatants and treated the supernatants for 30 min with 100 μL of LDH reaction solution. The OD value was measured by a microplate reader (Bio-Rad) at 490 nm. The percentage of LDH in cell lysates was calculated as the index of cellular injury.

### Apoptosis assay

The apoptotic cells were identified and measured using the Annexin V-FITC/PI apoptosis detection kit (Sigma-Aldrich, MO, USA) according to the manufacturer’s instructions. Briefly, cells at a density of 1 × 10^5^ cells/well were re-seeded in a 6-well plate. After treatment, cells were harvested and stained by 10 μL Annexin V-FITC and 5 μL propidium iodide (PI), followed by measurement with a BD FACSCalibur flow cytometer.

### Western blot analysis

Cells from experimental culture dishes were extracted using RIPA lysis buffer (Beyotime, Shanghai, China). Equal protein amounts (30 μg) were subjected to 10% SDS-PAGE and transferred onto PVDF membranes. The membranes were blocked with 5% skim milk diluted in TBS-Tween for 1 h and incubated at 4 °C overnight with anti-Bax (1: 500, #2774, Cell signaling), anti-Bcl-2 (1: 500, #2876, Proteintech), anti-Aven (1: 1000, #2865, Cell signaling) or anti-GAPDH (1: 500000, 10,494–1-AP, Proteintech). Next day, the membranes were incubated with HRP-conjugated secondary antibodies (1: 5000, SC-2005, Santa Cruz). An enhanced chemiluminescent (ECL)-Plus detection reagent (Santa Cruz, CA, USA) was used to detect the signal of protein expression with GAPDH as an internal control.

### Bioinformatics analyses and luciferase reporter assays

The online software TargetScan (http://www.targetscan.org/) was used to identify the potential target genes of miR-30b-5p. Among the list of target genes obtained, Aven, an apoptosis inhibitor, was predicted to have miR-30b-5p binding sites. For assessing Aven as a miR-30b-5p target, the pmirGLO reporter vector containing a wild type or mutant type miR-30b-5p binding site in the 3′-UTR of Aven (Aven WT or Aven MUT) was synthesized by Ribobio. The AC16 cells were cultured in 24-well plates and co-transfected with 50 ng of Aven WT or Aven MUT together with 20 μM miR-30b-5p inhibitor or NC using Lipofectamine 2000 (Invitrogen). Luciferase activities were analyzed after 48 h transfection on the Dual-Luciferase Reporter Assay System (Promega, Madison, USA).

### Statistical analysis

All quantitative data were analyzed by Prism V.5.0 software (GraphPad Software, California, USA) and presented as mean ± SD. Student’s t-test was used to compare the statistical difference between two groups. Comparisons of parameters among more than two groups were analyzed by one-way analysis of variance for a single factor. The value of *p* < 0.05 was considered statistically significant.

## Results

### MiR-30b-5p expression in AC16 cells under hypoxia

**Fig. 1:**
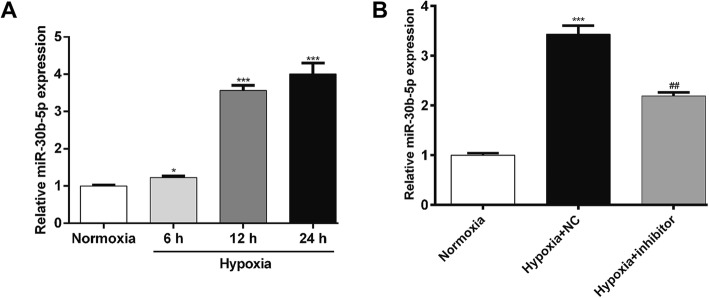
Inhibition of miR-30b-5p expression in hypoxic cardiomyocytes. **a** AC16 cells were exposed to hypoxia for 6, 12 and 24 h, respectively, and then harvested for analysis by quantitative real-time PCR. **b** The expression of miR-30b-5p was determined in AC16 cells transfected with miR-30b-5p inhibitor or NC, followed by 12 h hypoxia treatment. Cells cultured under normoxia were used as the control. *: hypoxia vs. normoxia; #: hypoxia + inhibitor vs. hypoxia + NC; **p < 0.01, ***p < 0.001, ##*p* < 0.01

AC16 was exposed to hypoxia to mimic the myocardial hypoxic injury in vitro. The expression level of miR-30b-5p was first determined in hypoxia-induced AC16 cells by qRT-PCR. As shown in Fig. 1a, the miR-30b-5p expression was significantly increased in AC16 cells after hypoxia compared with that in normoxia in a time-dependent manner. To further elucidate its biological function, we selected 12 h hypoxia and constructed a miR-30b-5p silenced cell model in vitro. As shown in Fig. 1b, the expression of miR-30b-5p was significantly downregulated in hypoxic AC16 cells by transfecting miR-30b-5p inhibitor (*p* < 0.01). Thus, a successful miR-30b-5p silenced cell model was constructed to investigate the biological function of miR-30b-5p in hypoxic cardiomyocytes.

### MiR-30b-5p silencing improved cell survival of hypoxic cardiomyocytes

**Fig. 2:**
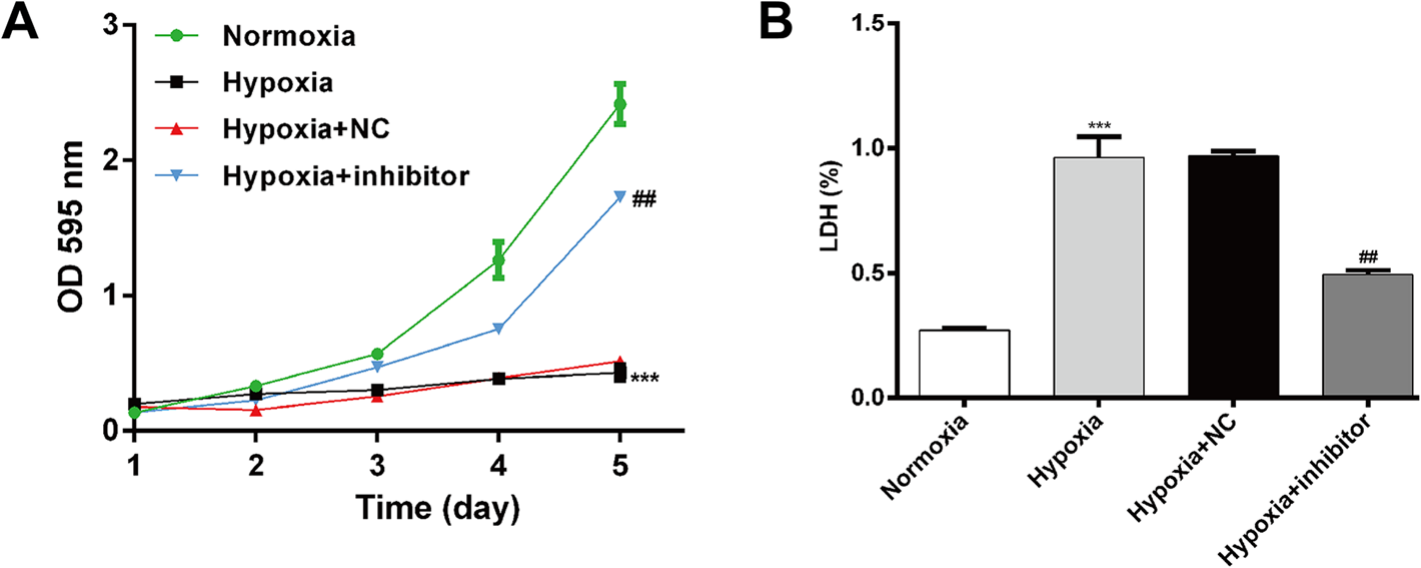
Inhibition of miR-30b-5p improves cell survival of hypoxic cardiomyocytes. AC16 cells were transfected with miR-30b-5p inhibitor or NC, followed by exposure to hypoxia for 12 h. Cell survival of cardiomyocytes was determined by MTT (**a**) and LDH (**b**) assays. LDH (%) means the percentage of LDH in cell lysates. *: hypoxia vs. normoxia; #: hypoxia + inhibitor vs. hypoxia + NC; **p < 0.01, ***p < 0.001, #*p* < 0.05, ##*p* < 0.01, ###*p* < 0.001

Next, we evaluated the effects of miR-30b-5p silencing on hypoxia-induced injury using MTT and LDH assay. As shown in Fig. 2a, hypoxia stimulation significantly impaired AC16 cell viability (*p* < 0.001), but it was apparently reversed by transfection with miR-30b-5p inhibitor (*p* < 0.01). The LDH assay (Fig. 2b) showed that hypoxia-induced cell injury was significantly attenuated by inhibition of miR-30b-5p. Collectively, our results demonstrated that miR-30b-5p silencing can protect cardiomyocytes from hypoxia-induced injury.

### MiR-30b-5p silencing inhibited hypoxia-induced apoptosis in cardiomyocytes

**Fig. 3:**
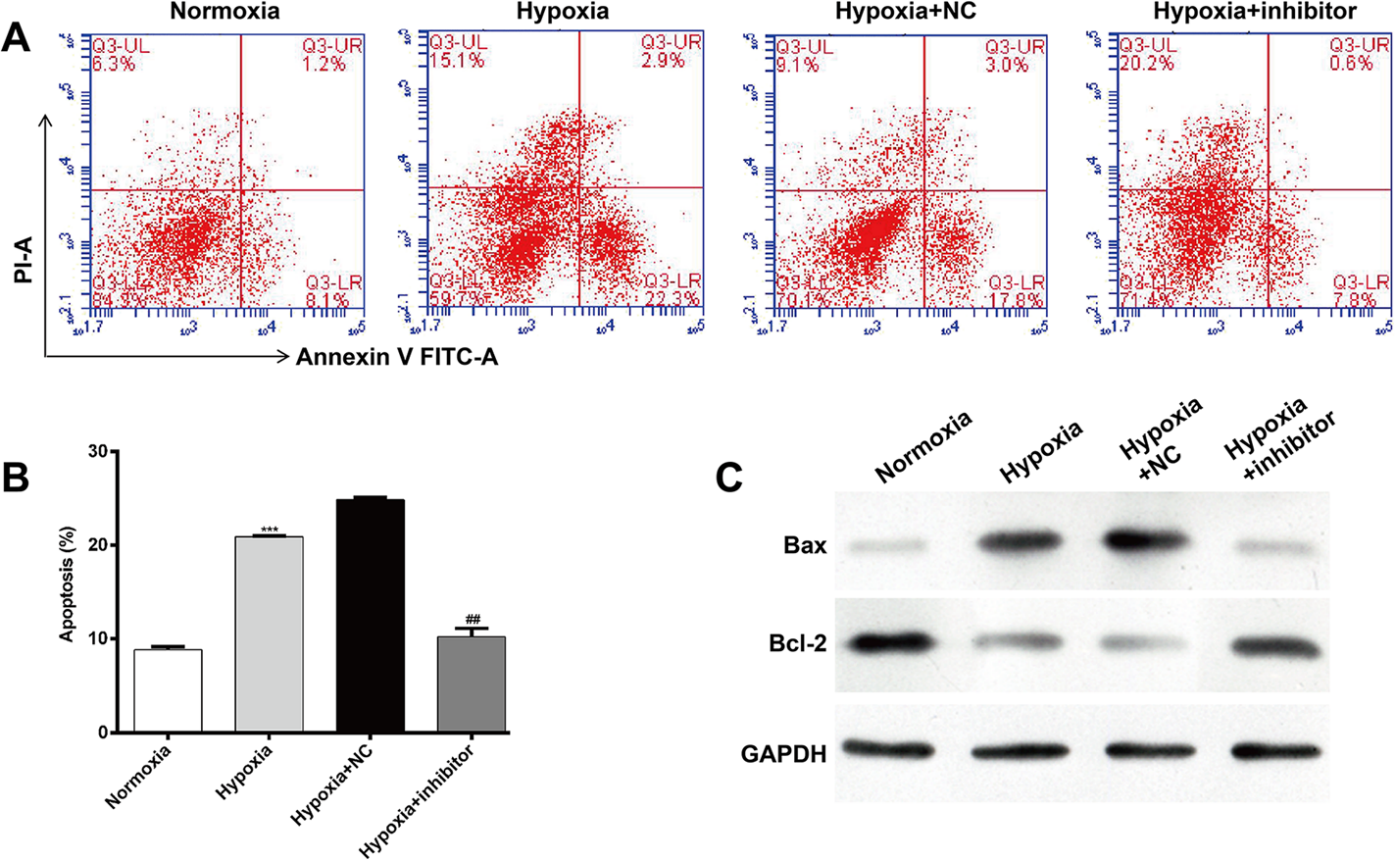
Inhibition of miR-30b-5p suppresses hypoxia-induced apoptosis in cardiomyocytes. **a** Representative captures of flow-cytometric data demonstrating the percent early apoptosis (Annexin V+/PI-) and late apoptosis (Annexin V+/PI+) in AC16 cells grown under normoxia or hypoxia with or without miR-30b-5p inhibitor. **b** Quantitation of A. **c** Protein expression of Bax and Bcl-2 was detected by western blot analysis in AC16 cells grown under normoxia or hypoxia with or without miR-30b-5p inhibitor. GAPDH was used as an internal control. *: hypoxia vs. normoxia; #: hypoxia + inhibitor vs. hypoxia + NC; ***p < 0.001, ##*p* < 0.01

Next, we used flow cytometry to evaluate the effect of miR-30b-5p inhibition on cell apoptosis in AC16. Representative captures of flow-cytometric analyses of cardiomyocytes grown under normoxia/hypoxia with or without miR-30b-5p inhibitor are depicted in Fig. 3a. Annexin V vs. PI plots from the gated cells showed the populations corresponding to viable and non-apoptotic (Annexin V−/PI−), early (Annexin V+/PI−), and late (Annexin V+/PI+) apoptotic cells and necrosis (Annexin V−/PI+). Further statistical analysis (Fig. 3b) demonstrated that exposing AC16 to hypoxia resulted in a significantly elevated apoptotic rate: 8.89 ± 0.34% vs. 21.27 ± 0.12% in normoxia vs. hypoxia (*p* < 0.001). However, miR-30b-5p inhibitor transfection significantly decreased cell apoptosis from 23.25 ± 0.21% to 11.30 ± 0.74% (*p* < 0.01). To further investigate the pro-apoptosis mechanism of miR-30b-5p, the expression levels of Bax and Bcl-2 protein were detected. As shown in Fig. 3c, an obvious decrease of Bax and apparent upregulation of anti-apoptotic protein Bcl-2 expression were observed in hypoxia-induced cardiomyocytes after inhibition of miR-30b-5p. Overall, these results suggest that miR-30b-5p silencing could suppress hypoxia-induced cardiomyocyte apoptosis.

### MiR-30b-5p directly targeted Aven by binding its 3′-UTR

**Fig. 4:**
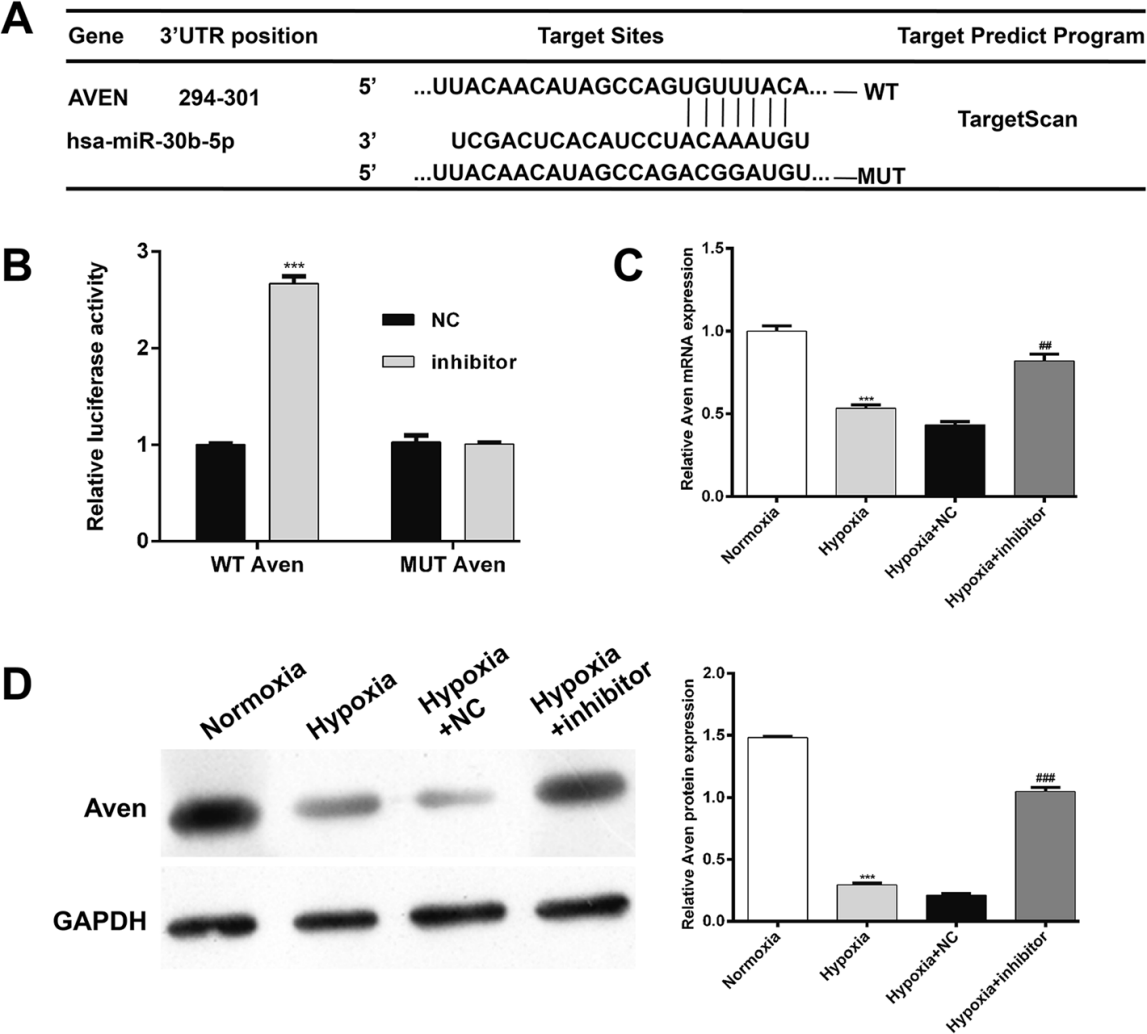
miR-30b-5p targets the 3′-UTR of Aven. **a** Sequence alignment of miR-30b-5p and 3′-UTR of Aven. **b** Dual-luciferase reporter assay. AC16 cells were co-transfected with miR-30b-5p inhibitor and a luciferase reporter containing the Aven 3′-UTR or mutant Aven 3′-UTR and incubated for 48 h. Relative luciferase activities were detected by the dual luciferase assay system. ***p < 0.001 vs. NC. The mRNA (**c**) and protein (**d**) expression levels of Aven were detected by qRT-PCR and Western blot analysis, respectively. *: hypoxia vs. normoxia; #: hypoxia + inhibitor vs. hypoxia + NC; ***p < 0.001, ##*p* < 0.01, ###*p* < 0.001

Aven was preliminarily identified as a putative target of miR-30b-5p by bioinformatics analysis (Fig. 4a). To verify this, a luciferase reporter assay was then performed in AC16 cells. The results revealed that downregulation of miR-30b-5p significantly elevated the luciferase activity of Aven-WT, but did not affect that of Aven-MUT (Fig. 4b, *p* < 0.001), implying that Aven was a target gene of miR-30b-5p. Moreover, the expression levels of Aven mRNA (Fig. 4c) and protein (Fig. 4d) were significantly reduced in hypoxia-induced cardiomyocytes, but obviously elevated after miR-30b-5p inhibitor transfection.

### Aven knockdown partially reversed the effects of miR-30b-5p silencing on cardiomyocytes under hypoxia

**Fig. 5:**
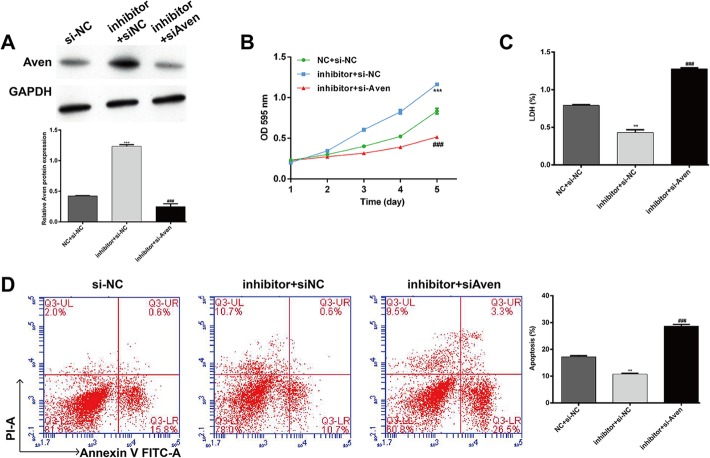
Knockdown of Aven partially reversed the protective effects of miR-30b-5p silencing. AC16 cells were co-transfected with miR-30b-5p inhibitor and siAven and then exposed to hypoxia for 12 h. **a** Protein expression levels of Aven were detected by Western blot analysis. Cell survival of cardiomyocytes was determined by MTT (**b**) and LDH (**c**) assays. **d** Cell apoptosis was evaluated by flow cytometry assay. *: inhibitor + si-NC vs. si-NC; #: inhibitor + siAven vs. inhibitor + si-NC; **p < 0.01, ***p < 0.001, ###*p* < 0.001

To investigate whether Aven was a functional regulator involved in the protective effects of miR-30b-5p inhibition against hypoxia, we performed rescue experiments in AC16 cells by co-transfection with miR-30b-5p inhibitor and si-Aven. As shown in Fig. 5a, Western blotting confirmed that the elevated expression of Aven caused by miR-30b-5p inhibition was significantly abrogated by Aven silencing. As expected, the protective effects of miR-30b-5p silencing against hypoxia-induced impaired cell viability (Fig. 5b), damage (Fig. 5c) and apoptosis (Fig. 5d) were markedly reversed by Aven knockdown in AC16. Collectively, these results further demonstrated that miR-30b-5p silencing could suppress hypoxia-induced injury by targeting Aven expression in cardiomyocytes.

## Discussion

Recently, tremendous effort has been made to reveal the action of miRNAs in human cardio-cerebrovascular diseases, including myocardial infarction. Here, we focus on the functional role of miR-30b-5p in cardiomyocytes under hypoxia. It has been reported that miR-30 family expression was enhanced in the murine model of myocardial infarction and hypoxia-induced cardiomyocytes [21] and restoration of miR-30b-5p suppressed cardiac hypertrophy via targeting CaMKIIδ [15]. As expected, miR-30b-5p expression was observed to be significantly elevated in cardiomyocytes under hypoxic conditions. Downregulation of miR-30b-5p alleviated hypoxia-induced cardiomyocyte injury, observed as increased cell viability, decreased LDH leakage, and a decreased apoptosis rate. Consistently, miR-30b-5p is correlated with physical activity-related improvements in vascular risk and remodeling [22]. Surprisingly, Aven was a target gene of miR-30b-5p and Aven knockdown showed a similar effect on cardiomyocytes. Our results suggest that upregulation of miR-30b-5p observed in cardiomyocytes under hypoxia possibly causally participated in the development of myocardial infarction.

According to the report from Sikorski et al. [23], miRNAs constitute the most extensively studied class of non-coding RNAs, which could initiate translational repression by recognizing specific target mRNA sequences within the 3′-UTR in mammalian cells. Hence, it is plausible that the miR-30 family may function as a regulator of cell life and death based on the specific cellular environments and their targets. For example, miR-30b impaired TRAIL-induced glioma cell apoptosis via suppressing the critical functional apoptotic protein caspase-3 [24]. Hyper-expression of miR-30b stimulates apoptosis and abrogates gastric tumor growth through binding to its recognition sites located in the 3′-UTR of plasminogen activator inhibitor-1 [25]. In hepatocellular carcinoma, targeting of AEG1 by miR-30a-5p results in inhibition of viability and cell proliferation, as well as acceleration of apoptosis [26]. Under hypoxia, we did verify that down-regulation of miR-30b-5p promoted cardiomyocyte proliferation and depressed apoptosis and LDH leakage, suggesting a protective role of miR-30b-5p in hypoxia-induced cardiomyocyte injury.

It is important to identify its target gene to reveal the molecular mechanism underlying miR-30b-5p function. Previous studies reported that proline-rich transmembrane protein 2 and CaMKIIδ are the targets for miR-30b-5p in glioblastoma and cardiac hypertrophy, respectively [15, 27]. Here, Aven was identified as a direct target of miR-30b-5p in cardiomyocytes under hypoxic conditions. Interestingly, Aven is a novel anti-apoptotic protein which is cleaved by cathepsin D to release its anti-apoptotic capability [28]. It has been well documented that AVEN could bind to Bcl-xl and Apaf-1, and function to interfere with the proteolytic activation of caspases [19]. Moreover, Chau et al. [19] also observed that Aven could interact with the anti-apoptotic Bcl-2 family member Bcl-2, but failed to interact with pro-apoptotic members such as Bax. Our study showed that Aven was a direct target gene of miR-30b-5p through miRNA-mRNA interactions. Moreover, we observed that Bax and Bcl-2 were respectively decreased and increased by knockdown of miR-30b-5p. These findings suggest that upregulation of Aven by miR-30b-5p knockdown caused increased Bcl-2 and decreased Bax, thus attenuating hypoxia-induced cardiomyocyte apoptosis.

## Conclusions

Overall, miR-30b-5p was found to regulate the cardiomyocyte injury including cell viability, LDH leakage and apoptosis. Inhibition of miR-30b-5p protected cardiomyocytes against hypoxia-induced cell damage through upregulation of AVEN. The present study expands our understanding of hypoxia-induced injury and survival of cardiomyocytes and may provide a clue for the exploration of a therapeutic strategy against myocardial infarction.

## Data Availability

All data generated or analyzed during this study are included in this published article.

## Abbreviations

ATCC: American Type Culture Collection
DMEM: Dulbecco’s modified Eagle’s medium
ECL: Enhanced chemiluminescence
FBS: Fetal bovine serum
LDH: Lactate dehydrogenase
qRT-PCR: Quantitative real-time PCR
